# Investigating the impact of insertion sequences and transposons in the genomes of the most significant phytopathogenic bacteria

**DOI:** 10.1099/mgen.0.001219

**Published:** 2024-04-03

**Authors:** Alexia Suellen Fernandes, Kiara França Campos, Jéssica Catarine Silva de Assis, Osiel Silva Gonçalves, Marisa Vieira de Queiroz, Denise Mara Soares Bazzolli, Mateus Ferreira Santana

**Affiliations:** 1 Department of Microbiology, Federal University of Viçosa, Viçosa, Brazil

**Keywords:** transposable genetic elements, phytopathogens, virulence and genome evolution

## Abstract

Genetic variability in phytopathogens is one of the main problems encountered for effective plant disease control. This fact may be related to the presence of transposable elements (TEs), but little is known about their role in host genomes. Here, we performed the most comprehensive analysis of insertion sequences (ISs) and transposons (Tns) in the genomes of the most important bacterial plant pathogens. A total of 35 692 ISs and 71 transposons were identified in 270 complete genomes. The level of pathogen–host specialization was found to be a significant determinant of the element distribution among the species. Some Tns were identified as carrying virulence factors, such as genes encoding effector proteins of the type III secretion system and resistance genes for the antimicrobial streptomycin. Evidence for IS-mediated ectopic recombination was identified in *Xanthomonas* genomes. Moreover, we found that IS elements tend to be inserted in regions near virulence and fitness genes, such ISs disrupting avirulence genes in *X. oryzae* genomes. In addition, transcriptome analysis under different stress conditions revealed differences in the expression of genes encoding transposases in the *Ralstonia solanacearum*, *X. oryzae*, and * P. syringae* species. Lastly, we also investigated the role of Tns in regulation via small noncoding regulatory RNAs and found these elements may target plant-cell transcriptional activators. Taken together, the results indicate that TEs may have a fundamental role in variability and virulence in plant pathogenic bacteria.

## Data Summary

Genome data analysed in this work are available in the National Center for Biotechnology Information database. The supplementary files, Synteny Fig. S1, available in the online version of this article, genomes utilized in this study (Table S1), elements identified with ISsaga (Table S2), elements identified with OASIS (Table S3), identified transposons (Table S4), and transcriptome (Table S5) are available on figshare https://doi.org/10.6084/m9.figshare.25413868.v1 [1].

**Impact Statement**

Diseases caused by phytopathogens pose a significant threat to agricultural production, resulting in yield losses in agriculture. Phytopathogenic bacterial diseases have increasingly become a challenging issue for control strategies. One hypothesis is that one of the roles played by mobile genetic elements, such as insertion sequences (ISs) and transposons (Tns), is in participating in genomic diversification events that allow the rapid evolution of plant pathogens by modulating characteristics such as virulence and pathogenicity. However, there are still some gaps that need to be explored, such as how these insertions in certain gene contexts directly reflect the pathogen–host interaction. In this study, we investigate the presence and distribution of ISs and Tns in the genomes of the top ten phytopathogenic bacteria infecting key crops, including *Pseudomonas syringae pathovars*, *Ralstonia solanacearum*, *Agrobacterium tumefaciens*, *Xanthomonas oryzae* pv. *oryzae*, *Xanthomonas campestris* pathovars, *Xanthomonas axonopodis pv. manihotis*, *Erwinia amylovora*, *Xylella fastidiosa*, *Dickeya* (*dadantii* and *solani*), and *Pectobacterium* (*carotovorum* and *atrosepticum*). Our results reveal a heterogeneous distribution of ISs within the genomes, with the abundance of these elements closely tied to the host’s life history. We also observe insertions near genes associated with transport systems, such as ABC efflux pumps and heavy metal transport proteins. Furthermore, we report the presence of Tns carrying virulence and resistance genes. Additional analyses indicate differential expression of these elements under environmental stress conditions, as well as the encoding of putative small RNAs. Thus, our findings provide evidence that transposable elements, such as ISs and Tns, may act as key drivers of genetic variability within the genomes of phytopathogens.

## Introduction

There are approximately 150 species of bacteria that cause plant diseases. These phytopathogens can infect a significant variety of crops, mainly of agronomic interest, and are considered the biggest threats to food security worldwide [2,3]. The major pathogenicity strategies used by bacteria to cause diseases in plants are well understood [4,6]. Plant pathogenic bacteria may cause different symptoms in their host, such as hyperplasia, spots, wilt, and tissue rot, among others [3]. However, despite the extensive knowledge about virulence mechanisms, many bacterial phytopathogens are difficult to control due to their high genomics plasticity. Still, some gaps need to be filled to help understand how pathogenicity mechanisms can promote the rapid adaptation of bacteria to different biotic and abiotic conditions. Transposable elements (TEs) stand out as important generators of genomic plasticity in this regard.

The main TEs found in bacteria genomes are the insertion sequences (ISs) and transposons (Tns). ISs are considered the simplest TEs with small sequences (fewer than 2.5 Kb) and usually only encode the genes for their mobility [7]. This element is classified into families based on the types of transposases and is flanked by short imperfect terminal inverted repeat sequences (TIRs) at their extremities [7,8]. By contrast, Tns are bigger and more complex, even despite sharing similar transposition mechanisms as ISs, being characterized by two inverted repeated sequences. Tns carry ‘passenger genes’ such as those for pathogenicity, adaptation to antibiotics, and resistance to heavy metals ([8,9] or virulence factors, such as the Tn*Xax1* transposon, which plays an important role in the pathogenicity of *Xanthomonas citri* [10].

The insertion of these elements into other genes, which affects the gene functions, is considered the main natural mutagens in genomes. The insertion of ISs and Tns may also impact regulatory regions or create chromosomal rearrangements [11]. Although most mutations resulting from transposition are considered negative, the presence and activity of TEs can be essential in the adaptation of these bacteria to the host, to different ecological niches, in the efficiency of pathogenicity mechanisms, in the colonization of new hosts, and the supplanting of resistance. Furthermore, many elements can be ‘domesticated,’ which means they can encode products of interest to the host cell and thus perform new functions [12,13].

Major phytopathogens are known to have a large number of TEs in their genomes [14,15]. However, little is known about the impact of ISs and Tns on adaptation and virulence in phytopathogenic bacteria. In this study, we aimed to identify, characterize, and perform an investigation of the ISs and Tns found in the genomes of the most significant phytopathogenic bacterial species worldwide according to the work of [16]. These plant pathogenic bacteria include *Pseudomonas syringae pathovars, Ralstonia solanacearum, Agrobacterium tumefaciens, Xanthomonas oryzae pv. oryzae, Xanthomonas campestris patovars, Xanthomonas axonopodis pv. manihotis, Erwinia amylovora, Xylella fastidiosa, Dickeya (dadantii and solani), Pectobacterium (carotovorum and atrosepticum*). Furthermore, we also investigated the involvement of TEs in ectopic recombination events, regulation via small RNAs (sRNAs), responses to different stress conditions and possible domestication.

## Methods

### Genomes and transcriptomes data

In total, 270 complete genomes representing the ten phytopathogenic bacterial species with the greatest scientific and economic impact in the world [16], were downloaded in fasta format from the National Center database for Biotechnology Information (NCBI – https://www.ncbi.nlm.nih.gov/assembly) in August 2020 (Table S1). RNAseq data belonging to the species, *Pseudomonas syringae isolate* MAFF212134, *Xanthomonas oryzae* KACC10331 and *Ralstonia solanacearum* GMI1000 (accession numbers PRJNA261679, PRJNA629827 and PRJNA671670, respectively) were downloaded in fastq format on the Sequence Read Archive (SRA) platform (www.ncbi.nl.nih.gov/sra).

### Identification and characterization of insertion sequences and transposons

Identification and localization of insertion sequences were performed using the programs Semi-Automatic IS Annotation (ISsaga) [17] and Optimized Annotation System for Insertion Sequences (OASIS) [15]. ISs were classified into families and subgroups according to the literature and *Everyman’s Guide to Bacterial Insertion Sequences* [7]. For the identification of the element direct target DNA repeats (DRs) and terminal inverted repeat sequences (TIRs), the Geneious 11.1.5 program was used.

The characterization of the Tns was done from the comparative analysis between the transposases and the terminal sequences of the predicted Tns against the sequence references deposited in the databases, ISfinder[18] and Prokaryotic Transposable Element Database and Web Portal for Transposon Analysis (Tncentral) [8]. The presence of the important fitness genes next to Tns and IS or inside of Tns was verified via blast (Basic Local Alignment Search Tool) of the ORFs against the databases, GenBank, Uniprot, Virulence Factors Database [19], Pathogen-Host Interactions database [20], Type III Secretion System Database [21], The Comprehensive Antibiotic Resistance Database [22], and ResFinder 4.0 [23].

### Phylogenetic inference

The first phylogenetic tree was constructed using sequences of the 16S rRNA gene from the genomes, and the second tree was constructed using Tn sequences found to determine whether Tns grouped according to their bacteria host. For that, we also included the previously identified Tns in *R. solanacearum,* Tn*6768*, Tn*6769*, Tn*6770*, Tn*6771*, Tn*6772* and Tn*6773* [24]. The alignment was performed via ClulstalW [25]. The phylogenetic trees generated from the sequences of the genes encoding the 16S rRNA of the bacteria and the Tns were obtained by the maximum-likelihood method, using general time reversible evolutionary as a model with 1000 bootstrap replicates. All steps were performed in MEGAX software [26] and phylogeny visualization and annotation were performed in the Interactive Tree of Life (iTOL) v4 interface (https://iTOL.embl.de/).

### Chromosomal rearrangements mediated by ISs and Tns

The visualization of potential rearrangements due to the presence of ISs and Tns was conducted through whole-genome synteny analysis, utilizing the Progressive Mauve software available in version 2.3.1 of Mauve [27]. For each species, two genomes were selected, one with a high number of these elements and another with a low count of ISs. IS and Tn sequences proximal to the predicted recombination regions were examined to search for recombination signals among elements that could indicate their role in the observed rearrangement. To accomplish this, recombination events were characterized using the RDP3 (Recombination Detection Program) software [28], which incorporates a set of seven distinct methods: Recombination Detection Program (RDP), GENECONV, 3Seq, Chimaera, Maximum Chi Square (MaxChi), SiScan, and BootScan. Only recombination events detected by at least four different methods were considered acceptable.

### Transcriptome analysis

Three RNAseq datasets belonging to the genomes of *P. syringae*, *X. oryzae* and *R. solanacearum* were analysed for the identification of differentially expressed TE sequences under stress conditions (Table S5a). Quality control of the sequences was performed using FastQC (v0.11.5) and Trimmomatic/0.36 software [29]. In the mapping step, reads were aligned with reference sequences, in this case, a representative of the subgroups of the predicted IS or Tn. Both mapping and comparison of expression levels were performed by Geneious 11.1.5 software according to the DESeq2 tutorial. The analyses of differentially expressed element sequences were performed by comparing the control condition and the treatments. Differential expression was calculated using Log2 Fold-Change (Log2FC>0, up-regulated) and (Log2FC 0, down-regulated), with a *P*-value 0.05. For the generation of the heatmaps, the values were plotted in the Interactive Tree of Life (iTOL).

### Prediction of regulatory sRNAs

Many ETs, throughout evolutionary time, are interrupted in the host’s genome in a process called domestication. These elements appear to play important roles including the selection of small regulatory sRNAs that may be involved in host gene regulation, copy number regulation and even transposition [30]. Therefore, for those elements that were altered in expression in the previous analysis, we decided to investigate the possibility of these transposable elements encoding small regulatory RNAs. All Tns sequences and IS sequences under positive selection pressure were evaluated for sRNAs prediction using four distinct methods: INFERNAL [31], RNAz [32], PresRAT [33] and Oasis 2 [34]. Only sRNAs that had the same target in the different algorithms and had sampled probability values equal to zero (CopraRNA *P*-value=0) were considered true. Functional annotation of the predicted target mRNAs was performed using two approaches. First, a manual keyword search using the gene name suggested by the CopraRNA program in the KEGG Orthology (KO) database. Second, by aligning predicted sequences against the Uniprot and Pfam (protein families database) databases.

## Results

### Distribution profile of insertion sequences in the phytopathogenic bacteria

**Fig. 1.**
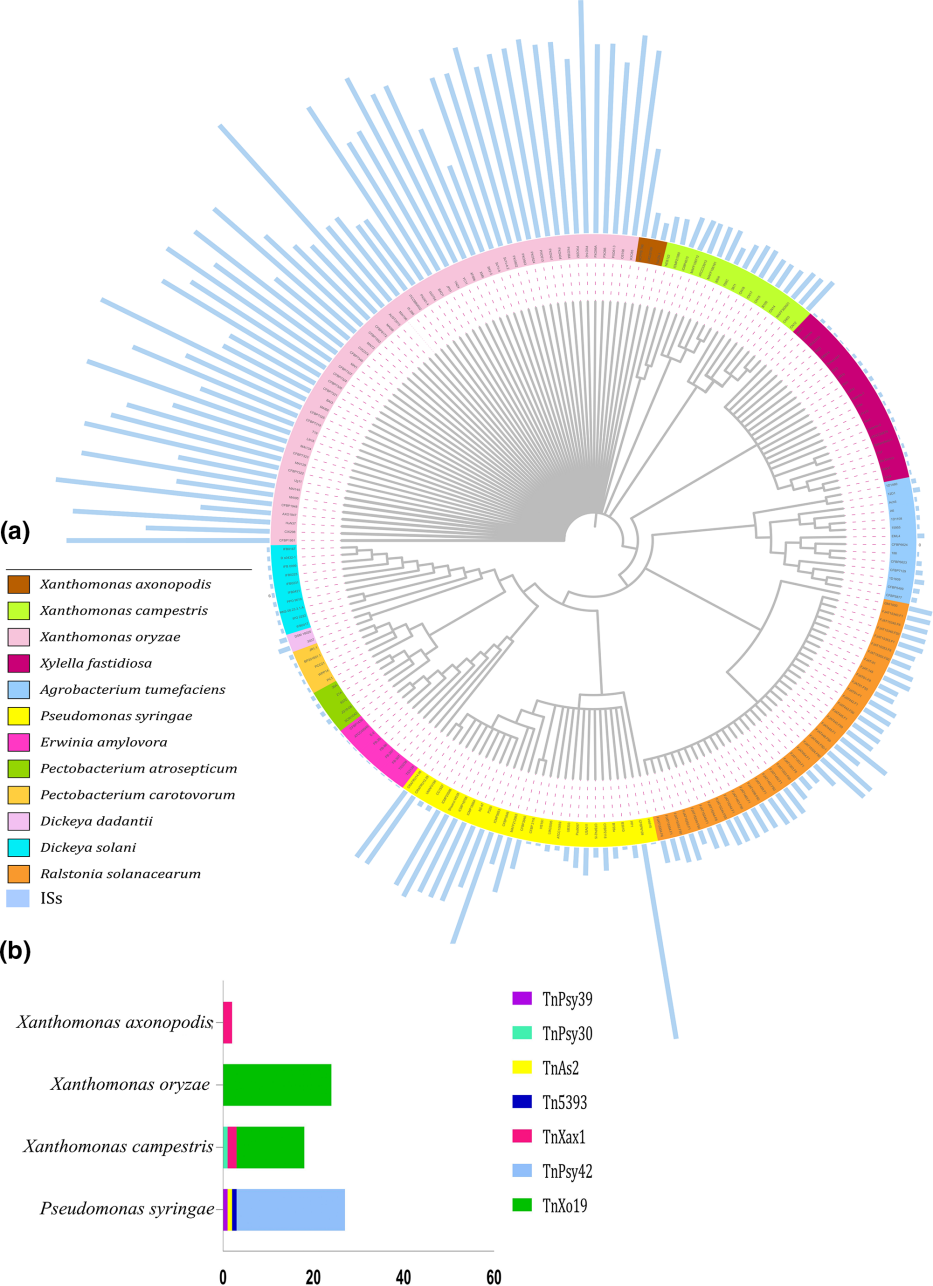
Distribution profile of insertion sequences and transposons found in the whole genomes analysed. (**a**) The phylogenetic tree was inferred by the neighbor-joining method using sequences of the gene encoding 16S rRNA from the 270 bacterial genomes investigated that are separated by colours. The bar graph around the tree indicates the number of predicted ISs elements for each genome. (**b**) The distribution and number of transposons found in the 270 genomes studied.

**Fig. 2.**
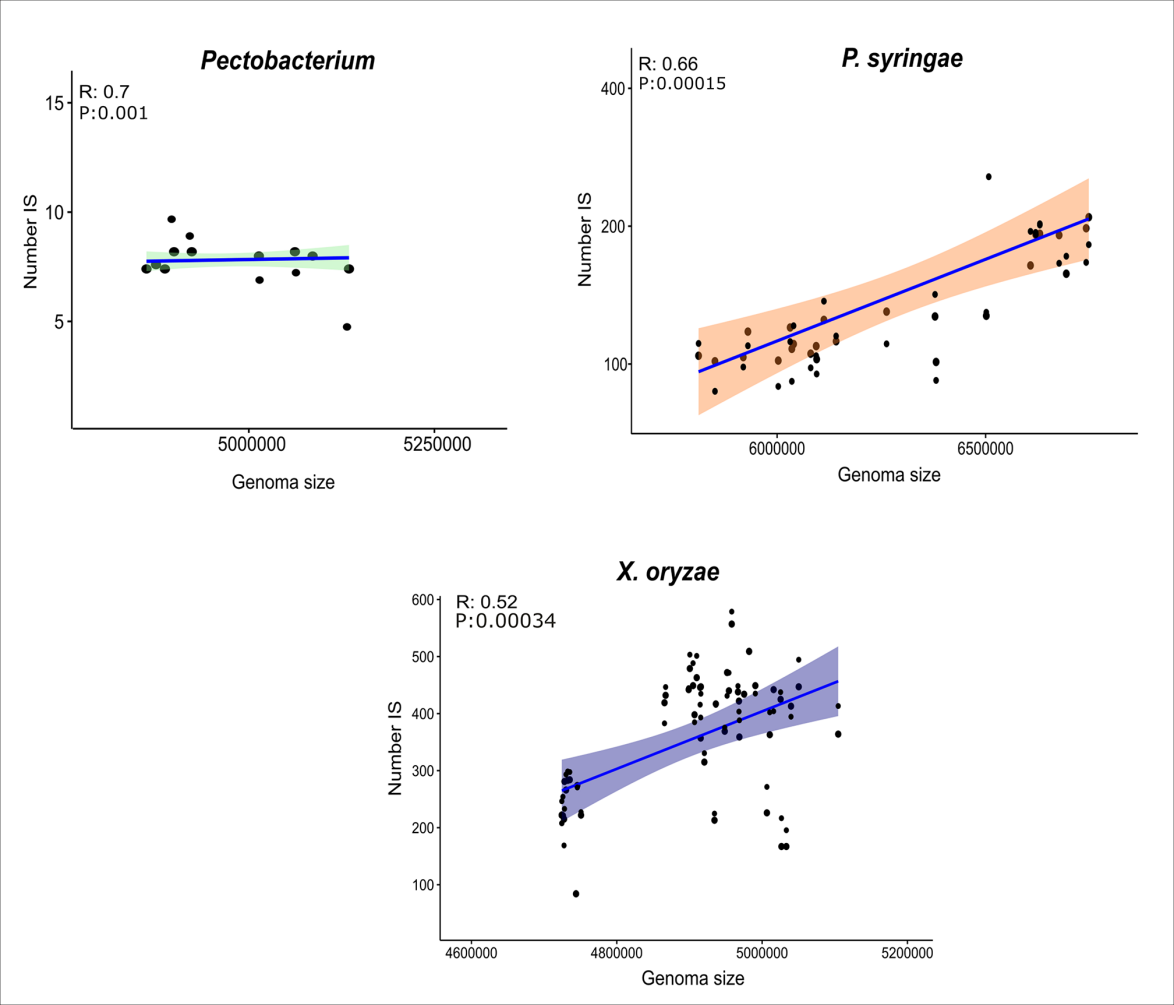
Graph of correlations between genome sizes and numbers of predicted IS of species that show significant correlations. The shaded regions correspond to the 95 % confidence interval based on the Pearson correlation coefficient.

We predicted 35 692 ISs in the 270 complete genomes examined (Tables S2 and S3). The distribution of ISs in the genomes of the phytopathogenic bacteria was quite heterogeneous, as some species have a higher copy number of these elements compared to others (Fig. 1a). Based on the number of predicted elements in each genome, a median was performed to estimate the number of insertion sequences for each species. In addition, we found 66 elements per genome in *P. syringae*, 60 in *R. solanacearum*, 10 in *A. tumefaciens*, 383 in *X. oryzae*, 65 in *X. campestris*, 60 in *X. axonopodis*, 2 in *E. amylovora*, 5 in *X. fastidiosa*, 21 in *D. dadantii* and 6 in *D. solani*, 6 in *P. carotovorum* and 8 in *P. astrosepticum*. Additionally, the presence of fragmented copies of ISs has also been observed in the genomes. Interestingly, the content of degraded IS elements in the species resembles the distribution of intact IS numbers, differing only in the case of *R. solanacearum*, which exhibited a lower number of degraded IS elements. However, two distinct groups can still be discerned, one characterized by a scarcity of fragmented ISs, with the median of predicted fragmented ISs shown in parentheses for each species: *E. amylovora* (3), *P. carotovorum* (5), *A. tumefaciens* (6.5), *X. fastidiosa* (7), *P. astrosepticum* (9.5), *D. solani* (9), *R. solanacearum* (10), *D. dadantii* (15.5); and another group with a higher number: *X. oryzae* (309), *X. campestris* (45), *X. axonopodis* (45), *P. syringae* (28) (Table S2). Overall, the IS families that had the highest number of representatives in the genomes were IS*5*, IS*3*, IS*701*, IS*1595*, and IS*630*. Although the majority of species lack the elements IS*5*, IS*701*, IS*630*, and IS*1595*, these elements were found in a high number of copies in the genomes of *X. oryzae*, where it is thought to be an expansion. Nevertheless, the IS3, IS110, and IS4 elements were found in most species genomes. With 5197 copies, IS3 stands out as the greatest element found in the majority of genomes, being absent only in *X. fastidiosa*. The IS3 elements comprise five subgroups that range from 1000 to 1750 bp in length and encode two partially overlapping ORFs. The IS5 element, present in seven species, has 13 102 copies and is subdivided into six subgroups ranging from 800 to 1500 base pairs (bp) with three subgroups identified in our analysis. IS4 and IS110 are the families having the most members among the ten phytopathogens, despite being absent from three species. Elements of the IS4 family range in size from 1400 to 1600 bp. It is an extremely heterogeneous family, but conserved structures help in distinguishing the subgroups. So far, seven members of this family, IS4, have been identified, while the IS110 family has only two recorded subgroups. Elements of the IS110 family have been detected in more than 130 bacterial species, ranging in size from 1200 to 1500 bp; it is the only family to carry a DEDD-like transposase and appears to have a specific insertion region [8]. On the other hand, IS701 has been identified in only two species, *R. solanacearum* and *X. oryzae*. However, only in *X. oryzae*, this element is present in 3537 copies, ranging from 1400 to 1550 bp, and encodes only one ORF. IS1595 was only found in *Xanthomonas* and *Ralstonia* genomes, comprising 1000 bp long with one ORF. An expansion of this element has been reported previously in *X. oryzae* [8]. Our data show that out of 3418 copies, 3295 belong to *X. oryzae*. Finally, we identified 2893 copies of IS630, which can have one or two ORFs and has approximately 1250 bp. In our data, this element was found in the top four bacteria. In addition to the five families discussed here, 15 other families are present (Tables S2 and S3). Although we expected that species of the same genus would show a similar pattern of IS families, our results showed that bacteria belonging to the genera *Xanthomonas* and pectinolytic bacteria such as *Pectobacterium* and *Dickeya* showed divergences between the types of families found and or in the number of copies of TEs in the different strains (Table 1). Moreover, none of the predicted families are found in all species, and some are only found in a particular organism, such as two unique copies of IS6 and IS607 detected only in *X. fastidiosa* and ISAs1 in *R. solanacearum*. Moreover, only the families IS*1*, ISH*3*, IS*1634*, IS*6*, IS*982*, IS*1380*, ISAzo*13*, ISH*6*, ISKra*4*, and ISLre*2* were not detected in the analysed genomes. In addition to the variations between the type families present in each species, we also detected variations in the number of elements found for each phytopathogen. It is visible that some bacteria, such as *X. oryzae* and *P. syringae*, have higher levels of elements than other bacteria, such as *E. amylovora* and *X. fastidiosa* (Fig. 1a; Table 1). In many organisms, especially eukaryotes, the increase in genome size is intrinsically related to the increase in the number or amount of transposable elements [35]. For our data, the abundance of IS elements was positively correlated with genome size for *X. oryzae*, *P. syringae* and *Pectobacterium* species (Fig. 2). We did not find a dependency relationship between the number of ISs found in the genomes and the size of the genomes for another nine species.

**Fig. 3.**
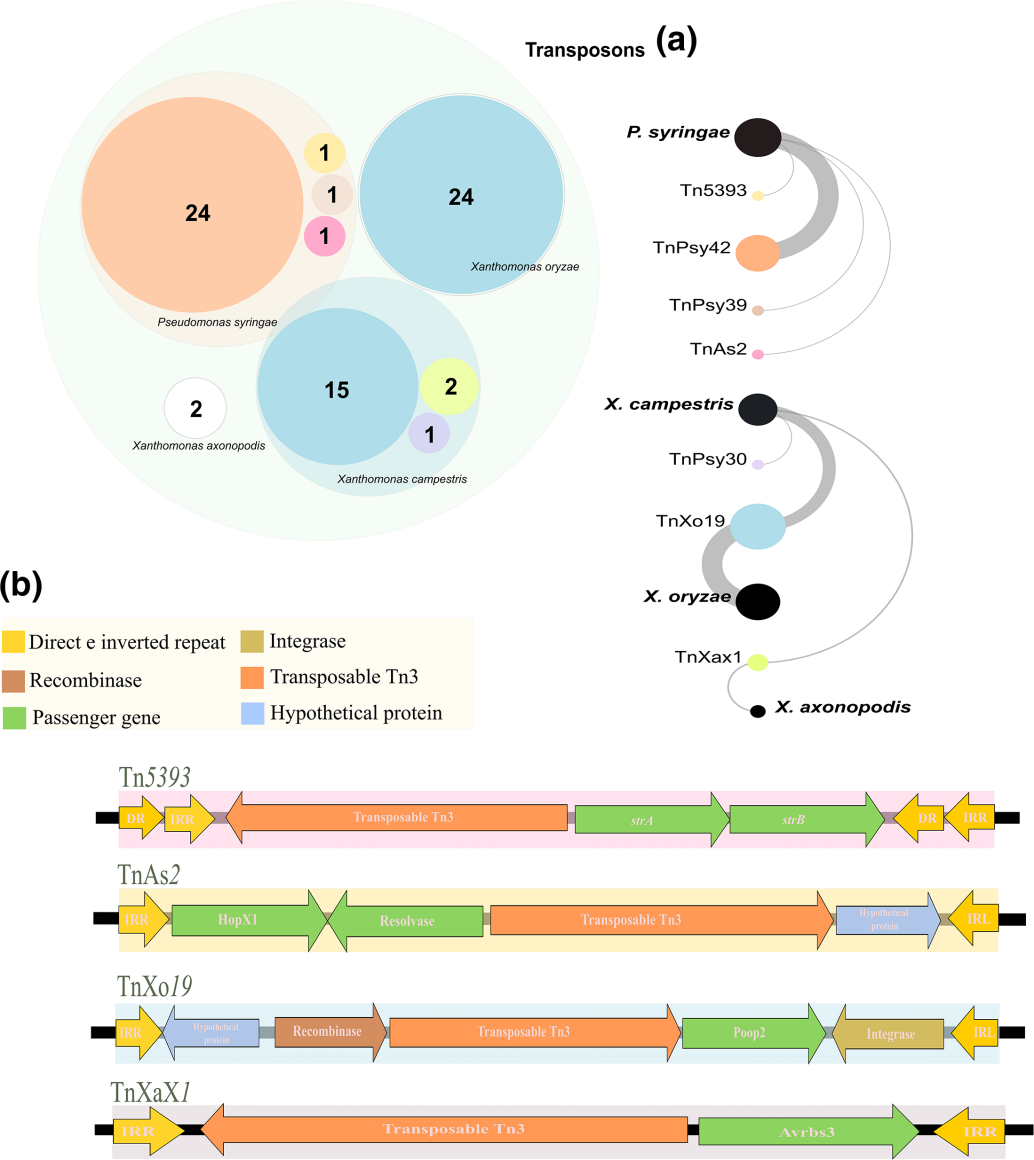
Representation of predicted transposons that may be carrying pathogenicity genes. (**a**) Total number of predicted transposons in each species. The circle on the left represents the set and quantity of each transposon found in each species, each coloured circle represents the total number of each predicted transposon, these can be identified in the figure on the right, which presents the names of each element. (**b**) Element Tn*5393* belongs to *Pseudomonas syringae* species, evidencing a pair of streptomycin resistance genes and TnAs*2* and TnXo*19,* which carry genes encoding effector proteins belonging to the type III secretion system and TnXax*1*, found in *X. campestris* isolates carrying a gene encoding an avirulence factor. (IRL) Left terminal, (IRR) right terminal, (*strA/strB*) streptomycin resistance genes, (HopX and PopP2) type III secretion effector proteins and (AvrBs3) effector protein.

**Table 1.** Number of copies of ISs present in each bacterial species investigated

Families	Ps	Rs	At	Xo	Xc	Xa	Ew	Xf	Dd	Ds	Pc	Pa	No. of ISs per family
**IS5**	322	597	13	1175	308	106	–	–	–	–	–	1	13 102
**IS3**	939	388	35	3178	453	131	22	–	19	20	8	4	5197
**IS701**	–	19	–	3518	–	–	–	–	–	–	–	–	3537
**IS630**	675	14	2	2192	–	–	–	–	–	–	–	10	2893
**IS30**	31	35	2	2493	–	–	–	–	–	–	–	–	2561
**ISL3**	16	67	–	1310	43	5	–	39	1	30	7	4	1522
**IS4**	234	386	32	631	61	6	–	–	2	–	2	2	1356
**IS256**	74	6	–	408	–	–	–	–	1	–	–	–	489
**IS1182**	384	14	1	–	–	–	–	–	–	–	1	1	401
**IS66**	158	86	15	31	–	–	–	–	–	–	–	–	290
**IS21**	192	89	6	1	–	–	–	–	–	–	–	–	288
**IS110**	65	71	13	37	4	–	–	–	29	10	2	2	233
**IS481**	8	1	2	191	–	9	–	–	–	–	2	3	216
**ISNCY**	–	20	6	3	1	–	–	–	–	8	10	3	51
**IS607**	–	–	–	–	–	–	–	35	–	–	–	–	35
**IS91**	14	–	–	3	3	–	8	–	–	–	–	6	34
**IS200/IS605**	–	–	–	–	–	6	–	2	6	–	–	–	14
**ISAs1**	–	1	–	–	–	–	–	–	–	–	–	–	1
**IS6**	–	–	–	–	–	–	–	1	–	–	–	–	1
**No. of ISs per species**	3112	1822	127	29 046	880	303	30	77	58	68	32	36	TOTAL

At
*Agrobacterium tumefaciens*
Dd
*Dikeya dadantii*
Ds
*Dickeya solani*
e Pa
*Pectobacterium atrosepticum*
Ew
*Erwinia amylovora*
Pc
*Pectobacterium carotovorum*
Ps
*Pseudomonas syringae*
Rs
*Ralstonia solanacearum*
Xa
*Xanthomonas axonopodis*
Xc
*Xanthomonas campestris*
Xf
*Xylella fastidiosa*
Xo
*Xanthomonas oryzae*

Furthermore, among the lineages of the species *X. campestris*, *E. amylovora*, *X. fastidiosa* and *D. solani* we observed insertion in a specific site in the same genomic context (Table 2).

**Table 2.** IS insertions in the same genomic context

Specie	Strain	Family	Context region	Gene
*X. campestris*	*8004*	*IS3 ssgr IS407*	*Biosynthesis of secondary metabolites*	cefD
*X. campestris*	*CN18*	*IS3 ssgr IS407*	*Biosynthesis of secondary metabolites*	cefD
*X. campestris*	*CN17*	*IS3 ssgr IS407*	*Biosynthesis of secondary metabolites*	cefD
*X. campestris*	*B100*	*IS3 ssgr IS407*	*Biosynthesis of secondary metabolites*	cefD
*X. campestris*	*CN03*	*IS3 ssgr IS407*	*Biosynthesis of secondary metabolites*	cefD
*X. campestris*	*MAFF302022*	*IS3 ssgr IS407*	*Biosynthesis of secondary metabolites*	cefD
*X. campestris*	*ICMP 4013*	*IS3 ssgr IS407*	*Biosynthesis of secondary metabolites*	cefD
*E. amylovora*	E-2	Tn3	Response abiotic stress	cpxP
*E. amylovora*	E-2	IS91	Response abiotic stress	yigB
*E. amylovora*	CFPB1430	Tn3	Response abiotic stress	cpxP
*E. amylovora*	CFPB1430	IS91	Response abiotic stress	yigB
*E. amylovora*	ATCC 49946	Tn3	Response abiotic stress	cpxP
*E. amylovora*	ATCC 49946	IS91	Response abiotic stress	yigB
*E. amylovora*	FB-86	Tn3	Response abiotic stress	cpxP
*E. amylovora*	FB-86	IS91	Response abiotic stress	yigB
*E. amylovora*	FB-207	Tn3	Response abiotic stress	cpxP
*E. amylovora*	FB-207	IS91	Response abiotic stress	yigB
*E. amylovora*	TS3238	Tn3	Response abiotic stress	cpxP
*E. amylovora*	TS3238	IS91	Response abiotic stress	yigB
*E. amylovora*	FB-307	Tn3	Response abiotic stress	cpxP
*E. amylovora*	FB-307	IS91	Response abiotic stress	yigB
*E. amylovora*	FB-20	Tn3	Response abiotic stress	cpxP
*E. amylovora*	FB-20	IS91	Response abiotic stress	yigB
*X. fastidiosa*	J1a12	Tn3	Metal cation efflux system	czcD
*X. fastidiosa*	Ann-1	Tn3	Metal cation efflux system	czcD
*X. fastidiosa*	3124	Tn3	Metal cation efflux system	czcD
*X. fastidiosa*	Pr8x	Tn3	Metal cation efflux system	czcD
*X. fastidiosa*	M23	Tn3	Metal cation efflux system	czcD
*X. fastidiosa*	Temecula1	Tn3	Metal cation efflux system	czcD
*X. fastidiosa*	Bakersfield-1	Tn3	Metal cation efflux system	czcD
*X. fastidiosa*	ATCC 35879	Tn3	Metal cation efflux system	czcD
*X. fastidiosa*	RH1	Tn3	Metal cation efflux system	czcD
*X. fastidiosa*	LM10	Tn3	Metal cation efflux system	czcD
*X. fastidiosa*	IVIA5901	Tn3	Metal cation efflux system	czcD
*X. fastidiosa*	Bakersfield-11	Tn3	Metal cation efflux system	czcD
*D. solani*	IOP2222	Tn3	Zinc transporter	zitB
*D. solani*	IFB 0099	Tn3	Zinc transporter	zitB
*D. solani*	RNS 08.23.3.1.A	Tn3	Zinc transporter	zitB
*D. solani*	IFB0421	Tn3	Zinc transporter	zitB
*D. solani*	IFB0231	Tn3	Zinc transporter	zitB
*D. solani*	IFB0417	Tn3	Zinc transporter	zitB
*D. solani*	IFB0167	Tn3	Zinc transporter	zitB

### The Tn distribution profile in the ten major phytopathogenic bacteria

**Fig. 4.**
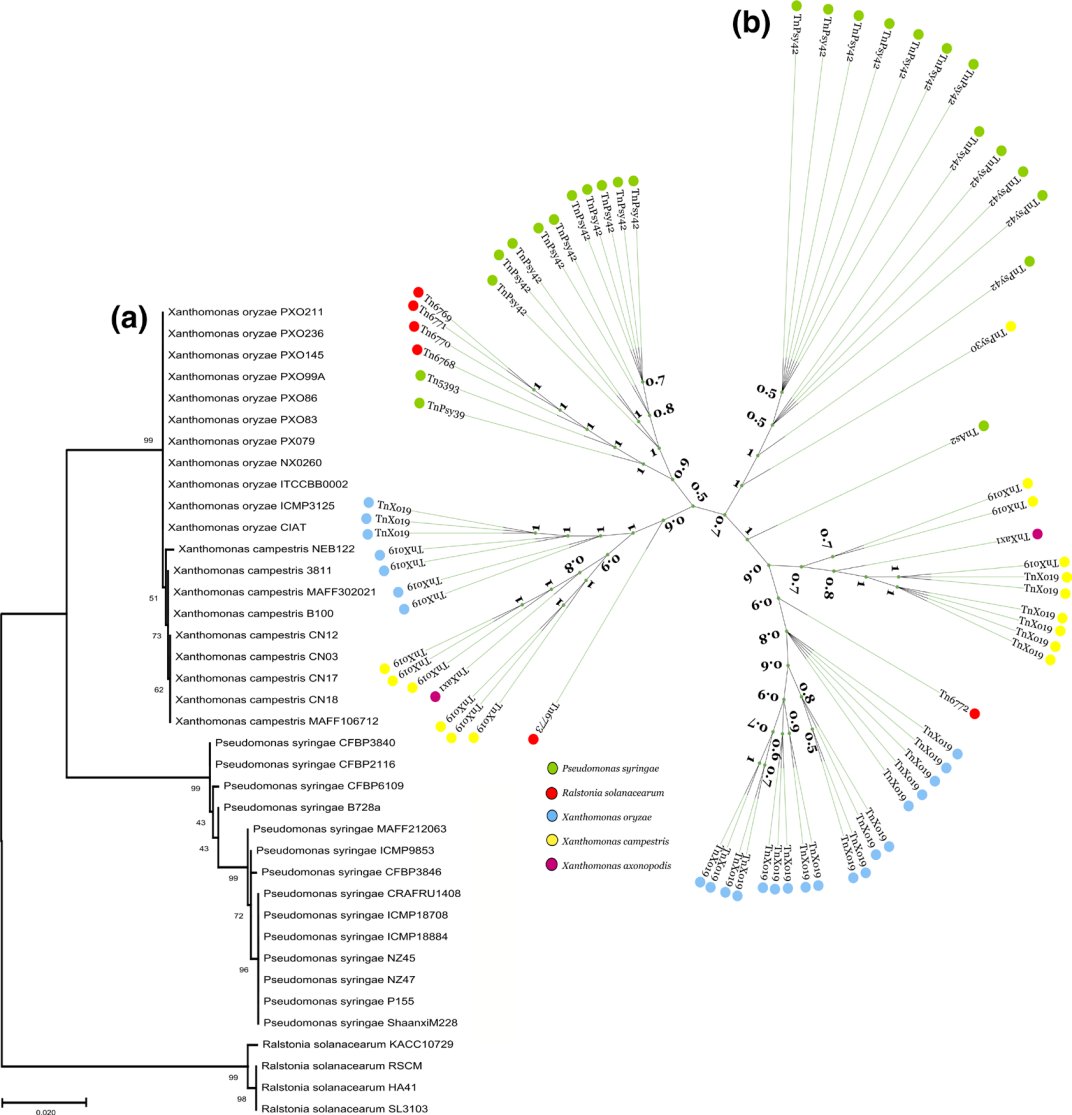
Phylogenetic interference. (**a**) Phylogenetic inference of isolates based on 16S rDNA gene sequences. (**b**) Phylogenetic tree of the complete copies of the transposons. The colours indicate the species in which the elements were identified. Both were constructed by inference of the maximum-likelihood method, taking into account the general time reversible evolutionary model.

The total number of transposons detected was 71, all belonging to the Tn3 familye (Figs1b  3a; Tables 3 and S4). Only four species, *P. syringae, X. campestris, X. oryzae*, and *X. axonopodis*, had transposons on chromosomes; the others were identified in plasmids. We mapped transposon elements carrying virulence and resistance genes (Fig. 3b), such as the Tn*5393* found in *P. syringae* (isolate B728a), this element encodes a Tn*3* family transposase and a pair of streptomycin resistance genes, *strA* and *strB*. Similarly, we found the TnAs*2* element in the genome of the *P. syringae* strain CFBP3846-1. Also, encoding a type Tn3 transposase, one resolvase and effector, hopX, belonging to the type III secretion system (TSS3). We similarly mapped the element TnXo*19,* belonging to the Tn3 family, in *X. campestris* CN03 carrying an effector Popp2. Furthermore, we detected the transposon, TnXax*1*, in the isolates CN17 and CN12 containing a gene encoding a transposase and carrying a gene encoding a protein member of the avrBs3 family, which has been identified as an effector and which may be involved in defence response mechanisms, preventing the host’s hypersensitivity response. According to the phylogenetic analysis, the Tn*3* family has largely clustered according to the species in which they are located. However, it was found that the element Tn*5393*, belonging to * P. syringae*, is more phylogenetically related to the group of elements of the species *R. solanacearum*. Similarly, the Tn*6772* element, present in *R. solanacearum*, clustering with elements of the species *X. oryzae*. In addition, the TnPsy*30* element in *X. campestris* is more phylogenetically related to elements of the species *P. syringae* (Fig. 4).

**Table 3.** Transposons found in the 270 complete genomes of major phytopathogenic bacteria

Species	Name	Family	No. found
*P. syringae*	Tn*5393*	Tn*3*	1
	TnPsy*42*	Tn*3*	24
	TnPsy*39*	Tn*3*	1
	TnAs*2*	Tn*3*	1
*X. campestris*	TnPsy*30*	Tn*3*	1
	TnXo*19*	Tn*3*	15
	TnXax*1*	Tn*3*	2
*X. oryzae*	TnXo*19*	Tn*3*	24
*X. axonopodis*	TnXax*1*	Tn*3*	2

### Chromosomal rearrangements mediated by ISs and Tns

We found evidence of ectopic recombination in the genomes of *X. axonopodis, X. campestris,* and *X. oryzae* (Fig. S1). One recombination event was identified for *X. oryzae* and *X. campestris*, and four events for *X. axonapodis*. The identification of recombination occurred only for elements belonging to the IS3 and IS5 families (Table 4).

**Table 4.** Evidence of potential recombination events detected

Specie	Family	Possible elements involved in recombination events	Detection method
*Xanthomonas axonapodis*	IS3	617140–618175,3436994–3438096,	GENECOV, BootScan, MaxChi, 3Seq
*Xanthomonas axonapodis*	IS3	402772–401670,4728014–4729 116	RDP, GENECOV,Chimaera,MaxChi, BootScan, SiScan, 3Seq
*Xanthomonas axonapodis*	IS3	617140–618175,4203695–4202593,836421–837523	RDP, GENECOV,Chimaera,MaxChi, BootScan, SiScan, 3Seq
*Xanthomonas axonapodis*	IS3	4203695–4202593,3436994–3438096	RDP, GENECOV,Chimaera,MaxChi, BootScan, SiScan, 3Seq
*Xanthomonas campestris*	IS5	2820068–2820874,2830209–2829229	RDP, GENECOV,Chimaera,MaxChi, BootScan, SiScan, 3Seq
*Xanthomonas oryzae*	IS3	498588–497486,	Chimaera, MaxChi, SiScan, 3Seq

### Analysis of differentially expressed elements

**Fig. 5.**
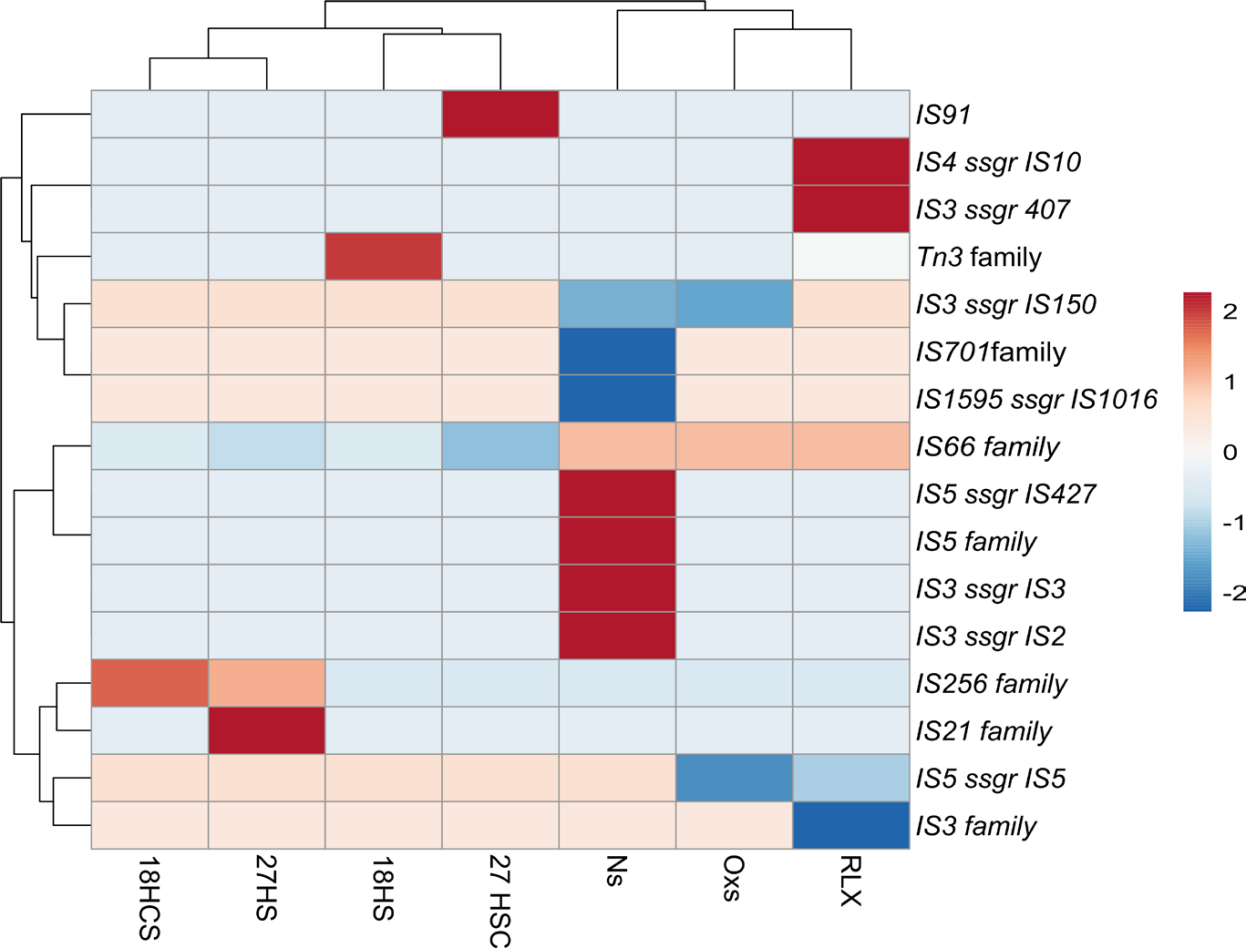
The expression level of each element is shown according to log2-fold values. Elements that had their transcription increased are shown in red and those that were suppressed are in blue. 18HS, 18HCS, 27HS, and 27HCS were cultured on coronatine-inducing media at temperatures of 18 and 27 °C. Nitric oxide and oxidative stress conditions, respectively. Culture of RLX in a pathogenicity-inducing medium.

We analysed whether the expression levels of the TEs under stress conditions may differ among ten elements belonging to the families Tn*3*, IS*66*, IS*21*, IS*256*, IS*91*, IS*3*, IS*701*, IS*1595*, IS*5* and IS*4* (Fig. 5 and Table S5). RNA-seq data were retrieved based on the experiment *P. syringae* exposed to culture conditions that used the coronatine-inducing media named HS and HSC at temperatures of 18 and 27 °C [36]. Our analyses found that there was a change in the expression of genes encoding transposases from some families, including Tn3, IS*256*, IS*21*, and IS*91*. By contrast, the expression of IS*66* was suppressed when exposed to higher temperatures, in all conditions compared to the control. Furthermore, according to our results, cultivation on LB medium compared to HS and HSC medium did not cause significant changes in the expression of the transposable elements evaluated.

In *X. oryzae*, the experiment sought to evaluate the differential expression profile when the bacterium was subjected to a culture medium with a pathogenicity inducer called RLX [37]. It was observed that there was a significant correlation between log 2-fold changes in expression levels that increased in the elements, IS*4*ssgrIS*10*, IS3ssgrIS*407* and suppressed in IS*3* family and IS*5*ssgrIS*5*. Furthermore, two copies of the IS*3*ssgrIS*407* element were found to be close to adaptation and virulence genes, one at 79 bp to a transporter family, MFS, and the other at 69 bp to a gene encoding an enzyme involved in plant tissue degradation, pectate lyase.

The differential expression analyses for *R. solanacearum* species were done using oxidative and nitric oxide stress conditions [38]. It was observed a greater number of ISs differentially expressed for the nitric oxide stress condition, such as IS3ssgrIS2, IS3ssgrIS3, IS5 and IS5ssgrIS427, which transcription was up-regulated, while in IS1595ssgrIS1016, IS3ssgrIS150 and IS701, these genes were down-regulated. Stress caused by lack of oxygen, on the other hand, led to decreased transcription of IS3ssgrIS150 and IS5ssgrIS5. In addition, we observed that IS3ssgrIS150, inserted near 100 bp of porin and at 79 bp of the operon PcaQ, had its expression downregulated with porin in nitric oxide shortage and PcaQ in oxygen shortage compared to the control.

### Small regulatory RNAs encoded by TEs

**Fig. 6.**
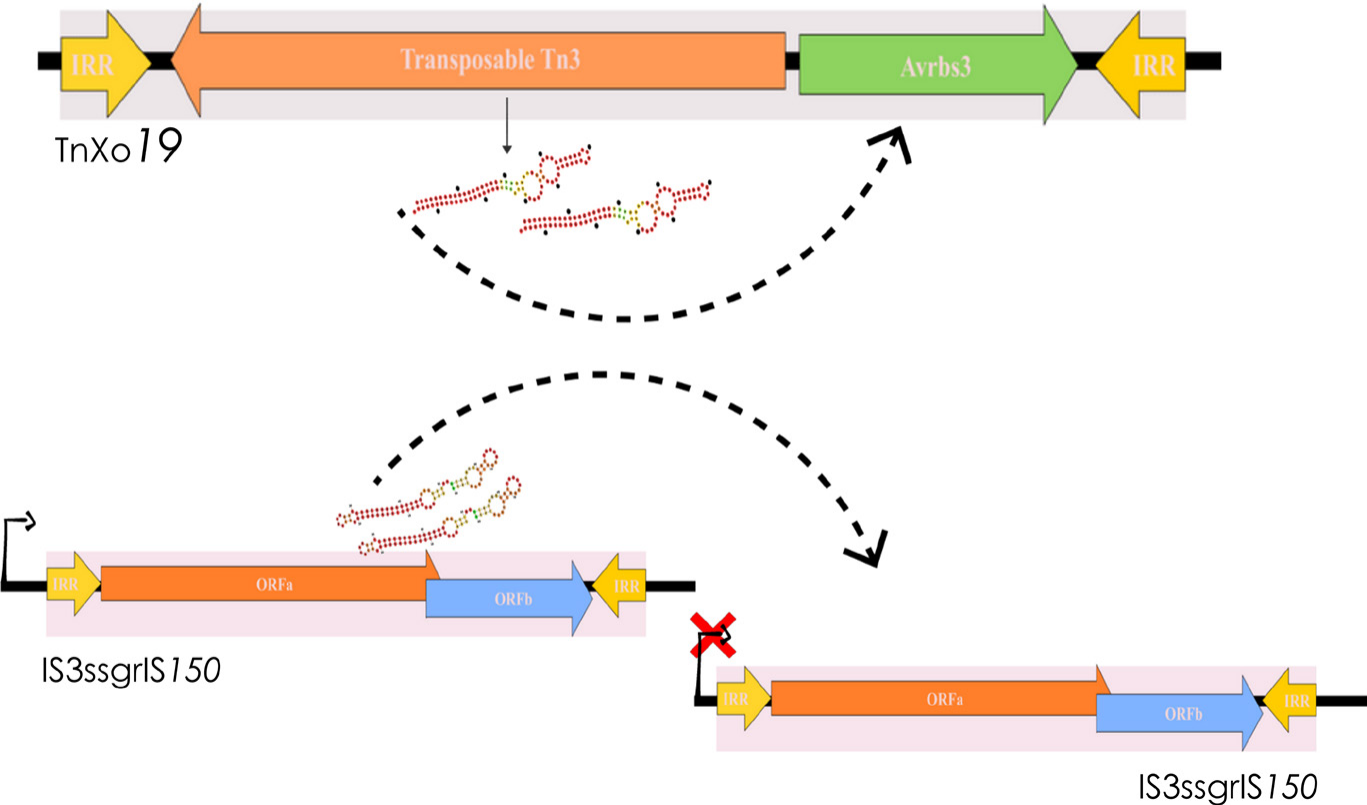
sRNA found by the transposable elements, IS*3*ssgrIS*150* and TnXo*19.* The predicted secondary structure was obtained by RNAfold. Above the figure, it is possible to visualize the element interacts with cognate targets.

Once we observed the difference in expression of some families due to changing environmental conditions, we performed an analysis to identify possible sRNAs that might be involved along the TEs. Thus, we observed that the IS3ssgrIS150 element in *R. solanacearum* GMI1000 encodes a predicted sRNA of 81 nucleotides, identified by all algorithms tested. The 28 homologues identified for this sRNA ranged from 70 to 90 nucleotides, five of which were predicted in the species *R. pseudosolanaceraum*. The identity level among the homologous was above 80%, and the predicted secondary structure (Fig. 6). Furthermore, we also performed the same analysis for the 71 transposons found in the analysed phytopathogenic bacteria. We found evidence of a possible sRNA encoded by transposon TnXo*19* belonging to *X. campestris* CN17 (Fig. 5). The sRNA predicted by three of the four methods used (INFERNAL, RNAz and PresRAT), has 85 nucleotides and was found in different pathovars of the genus *Xanthomonas*, with in total 115 homologues identified with identity greater than 80 %. The evaluation of potential regulatory targets of the predicted sRNAs revealed that the IS*3*ssgrIS*150* element interacts with cognate targets, and three copies of this element are found in the genome of *R. solanacearum* GMI100 (Table 5). The sRNA encoded by the TnXo*19* transposon, on the other hand, presented as possible target genes encoding plant-cell transcriptional activators, avrbs3, which is being carried by the element itself (Table 5).

**Table 5.** Targets predicted for transposable elements IS3ssgrIS150 and TnXo*19*

Specie	Strain	Target	Locus tag	Position mRNA	Position sRNA
***R. solanacearum***	GMI1000	IS3ssgrIS150	rs_rs11380rs_rs25525rs_rs02895	51 -- 122	1 -- 71
***X. campestris***	CN17	*avrBs3*	xcccn17_rs1113	10 -- 45	24 -- 60

## Discussion

In our work, we investigated for the first time the distribution profile of ISs and Tns present in major phytopathogenic bacteria of agronomic and scientific interest. Our results show a heterogeneous profile in the distribution of IS families among the phytopathogens. Overall, IS5 had a larger number of sequences in the genomes, however, IS3 was the family that was more widely distributed. Both IS5 and IS3 are families frequently found in prokaryote genomes [7]. In contrast, we were able to find elements specific to organisms such as IS6 and ISAs1. The abundance of elements varied considerably among the species. Interestingly, we observed that the abundance of copies of TEs varies with the lifestyle of the host. Bacteria that have a long evolutionary history on their hosts, such as *E. amylovora* or dependence as their vectors, such as *X. fastidiosa* and the genera *Dickeya* and *Pectobacterium* [39,40] tend to have a low accumulation of mobile elements in their genomes. Other studies also report this trend, for example, Wolbachia strains currently have a reduced genome with a low number of IS elements, but interestingly some strains still have a high number of TEs, which are interrupting pathways and which will probably be lost as part of the reduction and evolution of the genomes of these endosymbionts [41]. Thus, it is believed that without the immediate need for genomics adjustments for the survival of the pathogen, natural selection seems to act against the plasticity of the genome, since some functions no longer need to be performed by the organism [42,44]. On the other hand, bacteria that can replicate in the environment without a host, such as *P. syringae, A. tumefaciens, R. solanacearum*, and *Xanthomonas* spp. showed a significant number of entire ISs and ISs fragments ranging from 60 to 70 per genome, except in *A. tumefaciens*, which has an average of 10 elements per genome, and * X. oryzae* between 300 and 400 elements. This abundance of the ISs can be explained since these bacteria are existing in different niches, such as the phyllosphere and rhizosphere, and need to develop mechanisms to overcome abiotic and biotic conditions. Thus, the generation of plasticity from the gain of accessory genes, which confer functions associated with adaptation in the environment via horizontal transfer and new variations via recombination between genes mediated by ISs, may help the survival of these bacteria. This is well documented for some *P. syringae* and *X. campestris,* which are resistant to streptomycin-based pesticides due to the *strA*/*strB* gene pair in the acquired transposon, Tn*5393* [45].

*Xanthomonas oryzae* was the species that had the highest number of ISs per genome, ranging from 300 to 400 elements. Even though this species inhabits the phyllosphere, genetic heterogeneity among strains may be due to the variety of species and resistance genes encoded by various improved rice crops [46]. The genomic populational analysis showed the presence of TEs disrupting genes, performing recombination, and disseminating genes that confer fitness in the genomes of *X. oryzae*. As a result, the high number of pseudogenes in this species indicates recent evolution and that the species is in shift, speciation [46,47]. Another difference to our observations was that *A. tumefaciens*, the pathogen, showed ten elements per genome, despite being present in soil and not having specificity towards its host. However, this can be explained due to its genome being highly conserved, mainly due to its main mode of infection being quite specific [48,49].

Although numerically inferior, Tns can impact bacterial adaptation by carrying virulence and antimicrobial resistance genes, some carrying virulence and resistance genes. It becomes problematic because these genes can spread horizontally through mobile elements to other pathogens. For example, the transposon Tn*5393* was first reported in *E. amylovora*, but it has since been discovered in isolates of phytopathogenic bacteria including *P. syringae* and *X. campestris* [50]. We also identified two other transposons, TnAs*2* and TnXo*19*, containing sequences that encode type III secretion effector proteins, HopX and PopP2, in * P. syringae* (CFBP3846-1) and *X. campestris* (CN03), respectively.

Effector proteins of the T3SS are involved in pathogenesis, manipulating important host pathways, such as defence. The members of the HopX families are still not very well characterized, however, some research reports the participation of this protein in the induction of the defence response by hypersensitivity [51]. The PopP2 protein, on the other hand, is a member of an important family of effectors, YopJ, present in a wide range of bacterial pathogens, interestingly in phytopathogens, the PopP2 effector had been reported only in *R. solanacearum*, acting as an important inhibitor of gene expression related to plant defence [52]. Another type of effector gene used as a strategy for colonization in plants was verified in the analysed Tns, being named as TALEs (effector type activator of transcription). These act as transcriptional activators in plant cells, modulating the defence response in favour of the pathogen [53]. In *X. campestris* (CN17 and CN12), we detected a TnXax*1* transposon carrying a member of the TALE family, AvrBs3. This protein targets the resistance gene *Bs3* that is present in pepper species, and thus repetitive regions of AvrBs3 bind to promoter regions of the target gene (Bs3), preventing a hypersensitivity response [53].

In prokaryotes, the transfer of Tns and ISs between cells occurs via plasmid-mediated conjugation since these elements are abundant in this extrachromosomal molecule [54]. In this context, we observed that elements from different species were grouped into evolutionarily close clades. These results may represent a possible horizontal gene transfer between phytopathogenic bacteria of different species. This behaviour is already reported among several phytopathogens, as previously reported for Tn5393 [55]. Moreover, this phenomenon was also identified in an analysis by Assis *et al*. [56], in which the transposon TnXaj*417*, which confers copper resistance, was found in several species that cause plant diseases, such as those of the genera *Xanthomonas, Xylella*, and *Pseudomonas*. Interestingly, we showed that in each genus the TnXaj*417* underwent modifications, conserving only the *copA* and *copB* genes, thus suggesting that the acquisition of copper resistance was due to a recent HGT.

Furthermore, our results found evidence of recombination events mediated mainly by IS3 and IS5. These elements play an important role in adaptation for the species since the high transposition rate is the main diversification force of this pathogen [57]. Here, the species belonging to the genus *Xanthomonas* were the most abundant in terms of the number of TEs. Thus, the selection of six virulent strains of *X. oryzae* is believed to have occurred from the adoption of resistant rice varieties; however, in each population, the genome architecture is unique due to different forms of recombination mediated by mobile genetic elements [46].

TEs also participate in modulating host response during stressful situations. However, little is known about the effect of these elements in phytopathogens, so we used RNA-seq data to analyse the behaviour of insertion sequence families under stress conditions. Temperature changes in *P. syringae* between 18 and 27 °C interfered with the expression of some elements, where most of the element’s transcription increased. Furthermore, when *X. oryzae* isolates were subjected to a culture medium with a pathogenicity inducer, transposable elements close to MFS family transporters and pectate lyase increased their expression. Similarly, when placed in contexts of oxidative and nitrosative stress, we noticed that most elements had their expression increased when exposed to different nitric oxide conditions. Changing NO concentrations altered one of the main virulence factors in * R. solanacearum*, the T3SS effector proteins [38]. In isolates of this species analysed by [24], most IS elements were found inserted within genes encoding type III secretion effectors. Thus, the interference of nitrogen-associated metabolic pathways seems to interfere with the expression of virulence genes in pathogens. We also observed that ISs elements influence adjacent genes that are often associated with pathogenicity or the stress condition studied.

However, it is important to highlight that other studies have demonstrated that the change in the expression of ISs and adjacent genes can be triggered due to the expression of antisense RNAs encoded from intergenic or antisense regions of transposable elements, being capable of modulating gene expression by means of mechanisms such as the manipulation of mRNAs [58]. Thus, we understand that these elements appear to play an important role in host survival and participation during plant–pathogen interactions.

Finally, the families that had a differential expression in the different environmental conditions before were analysed in order to identify possible sRNAs that could be encoded. Small regulatory RNAs encoded by transposable elements have already been identified both in the Cis_sRNA form controlling the transposition of the element itself and also in the trans type regulating other genes [59,60]. We have identified two elements, IS3ssgrIS150 belonging to *R. solanacearum* and TnXo*19* belonging to * X. campestris*, that appear to be encoding putative sRNAs. In the case of the IS3ssgrIS150 element, the small RNA appears the IS, configuring itself as an sRNA-antisense, appears to be synthesized at the same locus as the IS. In the literature, many of the reported sRNAs that are encoded by TEs are Cis_sRNA type, and this mechanism acts to regulate transposition, thus controlling the copy number of the elements in the genome [61]. On the other hand, the sRNA encoded by TnXo*19* present in the *X. campestris* genome seems to have a different biological role. According to our data, the transposase referring to the Tn3 family is responsible for the transcription of the sRNA, which targets an effector protein, AvrBs3, belonging to a family of plant transcriptional activators called TALEs. Thus, we propose that the sRNA can regulate avrBs3 gene transcription during plant–pathogen interaction, maximizing *X. campestris* virulence since these proteins bind to plant resistance gene promoters, resulting in host susceptibility.

Taken together, in this work we performed the largest and most complete prediction of transposable elements in the genome of phytopathogenic bacteria. The abundance of these elements in the genome seems to be related to the type of infection mechanism and the level of pathogen–host specialization. We have demonstrated the involvement of TEs in ectopic recombination events, their expression under stress conditions, and their involvement in the regulation of sRNAS, demonstrating that these elements may play roles in genetic variability and, consequently, in pathogenesis. Thus, we believe that understanding the mechanisms of pathogen variability mediated by transposable elements may in the future aid in new approaches to plant disease control.

## supplementary material

10.1099/mgen.0.001219 Uncited Supplementary Material 1.

## Abbreviations

DR: direct repeat
HGT: horizontal gene transfer
IS: insertion sequence
MGE: mobile genetic element
NCBI: National Center for Biotechnology Information
SRA: Sequence Read Archive
sRNA: small regulatory RNAs
TE: transposable element
T3EP: type III effector protein
TIR: terminal inverted repeat
Tn: transposon
