# Supplementary material for: Investigating the impact of insertion sequences and transposons in the genomes of the most significant phytopathogenic bacteria
Source: Microb Genom. 2024 Apr 3;10(4):001219. doi: 10.1099/mgen.0.001219 (PMC11092175; doi:10.1099/mgen.0.001219)
Supplement: Uncited Supplementary Material 1. [file mgen-10-01219-s001.pdf]

Supplementary material

Link access:

https://drive.google.com/drive/folders/1LtKxf9MXFrs86-maLyNyaiQaQ8Nf9jI_?usp=share_link
